# Dynamic cystoscopy to optimize preoperative assessment of bladder endometriosis

**DOI:** 10.1590/S1677-5538.IBJU.2022.0594

**Published:** 2023-05-29

**Authors:** Fernando Salles da Silva, Luciano Alves Favorito, Claúdio Peixoto Crispi, Marlon de Freitas Fonseca, José Anacleto de Resende

**Affiliations:** 1 Universidade do Estado do Rio de Janeiro Rio de Janeiro RJ Brasil Universidade do Estado do Rio de Janeiro - Uerj, Rio de Janeiro, RJ, Brasil;; 2 Unidade de Pesquisa Urogenital Universidade do Estado do Rio de Janeiro Rio de Janeiro RJ Brasil Unidade de Pesquisa Urogenital, Universidade do Estado do Rio de Janeiro - Uerj, Rio de Janeiro, RJ, Brasil;; 3 Instituto Crispi Rio de Janeiro RJ Brasil Instituto Crispi, Rio de Janeiro, RJ, Brasil;; 4 Instituto Fernandes Figueira Rio de Janeiro RJ Brasil Instituto Fernandes Figueira, Rio de Janeiro, RJ, Brasil

**Keywords:** Endometriosis, Urinary Bladder, Cystoscopy

## Abstract

**Purpose:**

Bladder endometriosis (BE) accounts for 84% of cases of urinary tract involvement. The use of cystoscopy for preoperative evaluation is limited. The aim of this study was to evaluate the accuracy of preoperative dynamic cystoscopy (DC) in patients undergoing surgery for deep endometriosis and to describe the main findings and their impact on surgical planning.

**Materials and Methods:**

This cross-sectional observational study was conducted from January 2011 to March 2022. DC findings were divided into two groups according to the depth of involvement. To estimate sensitivity, specificity, positive predictive value (PPV) and negative predictive value (NPV), laparoscopic findings of bladder involvement and histopathological report were used as the gold standard.

**Results:**

We included 157 patients in this study. 41 had abnormalities in DC. Of these, 39 had abnormalities that were confirmed intraoperatively. The sensitivity and specificity of the test were 58.21% and 97.78%, respectively. PPV was 95.12%, and NPV was 75.86%. The presence of any lesions in the DC had a diagnostic odds ratio (OR) of 61.28 for BE. Patients with BE type 2 had a higher rate of partial cystectomy than those with BE type 1 lesions (OR 9.72 CI 95% 1.9-49.1)

**Conclusion:**

DC appears to be a highly specific test with lower sensitivity. DC abnormalities are associated with a higher ratio of bladder surgery for the treatment of deep endometriosis, and BE type 2 seems to be associated with a greater ratio (9.72) of partial cystectomy.

## INTRODUCTION

Endometriosis is a chronic condition defined by the presence of endometrial tissue outside the uterus ( 1 ). Retrograde menstruation of endometrial cells is believed to promote their implantation into the peritoneum, resulting in inflammation and fibrosis ( 2 ). Endometriosis affects 6-10% of women of reproductive age ( 3 ), and lesions can be of three types: superficial lesions, ovarian endometriomas, or deep endometriosis (infiltration greater than 5 mm in depth) ( 1 ). The urinary tract is affected in 1-2% of cases, and bladder endometriosis (BE) is the most common presentation (84% of these cases), mainly in the dome and bladder base ( 4 ).

Bladder involvement can be primary when it occurs spontaneously and secondary when it results from a previous pelvic procedure, such as hysterectomy. It is associated with injuries in other locations in 90% of the cases ( 4 ). There are two distinct pathophysiological mechanisms of BE. The dome is affected with the development of implants in the cul-de-sac. Trigonal lesions are secondary to anterior wall adenomyosis ( 5 ). Urinary symptoms may occur when deep endometriosis affects the lower urinary tract and adjacent structures. Parametrial involvement is associated with urinary voiding symptoms, whereas bladder infiltration is mainly associated with storage symptoms ( 6 ).

Transvaginal ultrasonography (TVUS) may be considered the first-line technique for the diagnosis of BE ( 4 ) and has a specificity of almost 100%; however, it is worse when the lesions are smaller than 3 cm or if the patient has already undergone surgical procedures. In these cases, the sensitivity did not exceed 50% ( 7 ).

Magnetic resonance imaging (MRI) may also be used for the evaluation of BE; however, there are no well-established protocols for its performance ( 7 ). MRI sensitivity reached 88%, and specificity was greater than 98% ( 8 ).

Cystoscopy may be particularly important in patients in whom MRI or TVUS findings suggest endometriosis in the anterior compartment and may define the exact location of the lesion, size, and distance to the ureteral ostia as well as its projection along the intramural ureter ( 4 ).

Although TVUS and MRI have a role defined in the preoperative evaluation with a good level of evidence, data on the use of cystoscopy in this scenario are scarce, with small samples and no established accuracy ( 4 , 9 ). Dynamic cystoscopy (DC) differs from conventional cystoscopy in that it combines bimanual palpation.

Our hypothesis is that DC can be useful in the preoperative evaluation of deep endometriosis, and we aimed to determine the accuracy of this diagnostic method, in addition to describing the main findings in the evaluation.

## MATERIAL AND METHODS

The protocol was approved by the Committee for Ethical Human Experimentation of our university and was carried out in accordance with the ethical standards of the hospital’s institutional committee on human experimentation (IRB number 30732420.3.0000.5259). This manuscript is based on the STARD statement ( 10 ).

This was a cross-sectional observational study, with prospective preplanned data collection to evaluate the accuracy of preoperative DC in a series of patients undergoing minimally invasive surgery for the treatment of deep endometriosis between January 2011 and March 2022. All included patients were treated at reference centers for assistance and research on patients with deep endometriosis. Therapeutic conduct followed the guidelines of the American Society of Reproductive Medicine (ASRM) and the European Society of Human Reproduction and Embryology (ESHRE) ( 11 ).

Inclusion criteria were women with deep endometriosis in the anterior, middle, or posterior compartment who underwent preoperative DC. It is important to highlight that the use of this method changed in this historical series. Initially, in the first years around 2011, this test was used in all patients preoperatively. After the second half of 2017, DC had a more restricted use, mainly limited to patients with suspected involvement of the anterior compartment of the pelvis. In recent years, since 2019, its use has become even more limited, being only performed in patients with suspected bladder involvement on MRI.

Exclusion criteria were patients who referred some condition that, although not associated with endometriosis or its treatment, were evidently liable to confuse the analysis, such as previous pelvic or perineal surgeries, urological or not (except for normal and uncomplicated cesarean deliveries), women who had already undergone pelvic radiation, and those with pelvic organs prolapses.

### Dynamic cystoscopy (DC)

Cystoscopy under sedation was performed using a Storz® 17-French rigid cystoscope and a 30-degree objective lens. In conventional cystoscopy, the bladder is systematically evaluated under maximal irrigation with saline solution. Initially, the floor of the bladder and trigone are surveyed. The ureteral orifices are noted, and the remainder of the bladder wall are inspected ( 12 ).

**Figure 1:**
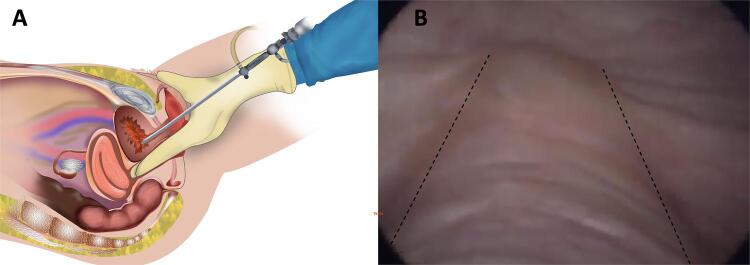
Dynamic cystoscopy (DC).

We denominated DC as the evaluation performed concomitantly with a physical examination of the pelvis. Associated vaginal and bimanual palpation allows evaluation of adhesions, retractions, and reduced bladder mobility ( Figure-1A ).

1A) Illustration demonstrating the procedure, which consists of a conventional cystoscopy associated with vaginal palpation. 1B) Vaginal palpation (dashed line) allows to identify and delimit a deep lesion in the bladder (type 1 lesion), nontender and fixed, which could go unnoticed as a roughness in its wall in a conventional cystoscopy.

Concomitant palpation also allows the location of possible deep nodules in the posterior bladder wall, noticed as indurated and immobile lesions, which cannot be identified by conventional cystoscopy ( Figure-1B ).

### Dynamic cystoscopy classification of bladder involvement

**Figure 2:**
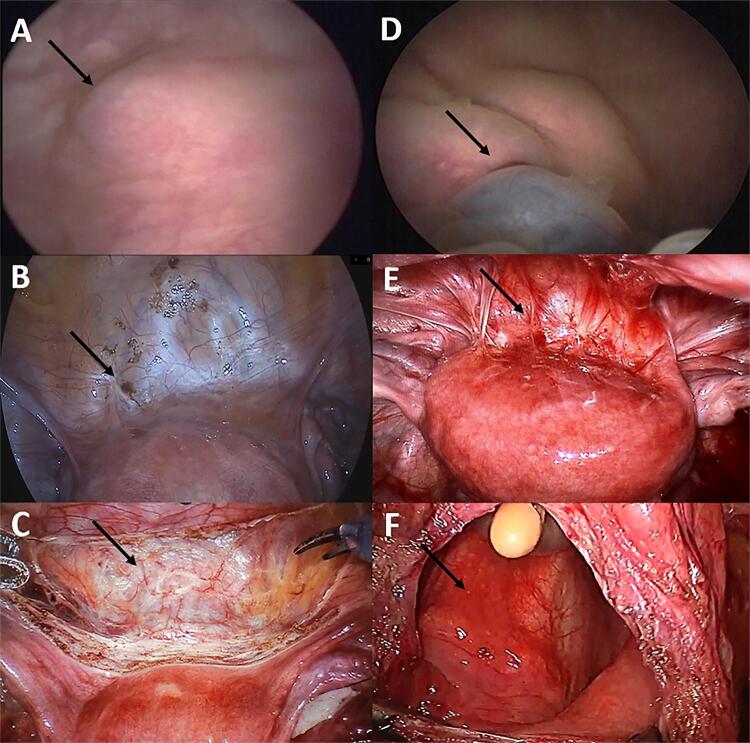
The figure shows 2 different cases of bladder endometriosis (BE). A, B, C) Thirty-two-year patient with BE saving the inner layer.

We classified the abnormal DC findings in two types: Type 1 lesions included extrinsic nodules in the bladder wall that did not extend beyond the muscular layer and were seen only as a protrusion adhered to the bladder wall. Type 2 lesions affected the mucosa and were identified as typical adenomatous red or bluish masses on the inner surface ( Figure-2 ).

Dynamic cystoscopy (DC) evidencing BE type 1, a nodule adhered to the bladder wall, without mucosal involvement (arrow). B) Laparoscopic view of the anterior compartment of the female pelvis. Minor bladder involvement in the peritoneal surface of the bladder (arrow). C) Treatment of BE in a more conservative resection- bladder shaving (arrow). D, E, F) -thirty three- year patient with BE involving bladder mucosa. D) DC evidencing BE type 2, an infiltrative lesion involving bladder mucosa with a typical blush mass (arrow). E) Laparoscopic view of the anterior compartment of the female pelvis with extensive adhesions and endometriotic lesions on the surface of the bladder (arrow) F) Treatment of BE: partial cystectomy, with opening of the mucosa (arrow).

### Assessing accuracy of dynamic cystoscopy (DC)

To assess the accuracy of the DC, sensitivity, specificity, positive predictive value (PPV), and negative predictive value (NPV), laparoscopic findings of bladder involvement were used as the gold standard. When endometriosis was detected through laparoscopic observation, the lesions were resected, and histopathological analysis was performed to confirm the diagnosis in all surgical specimens.

Laparoscopic findings considered abnormal included peritoneal disease on the bladder surface or infiltrative deep endometriosis that reached the muscular layer or bladder mucosa. The surgical technique for the treatment of bladder endometriosis involves partial cystectomy, defined as complete resection of the bladder wall in the affected region, or bladder shaving, an adapted technique for the treatment of intestinal endometriosis that consists of superficial resection of the organ, preservation of the mucosa, and maximum disease-free wall thickness ( Figure-2 ) ( 13 ).

Disease confined to the vesico-uterine septum was not considered BE, and this involvement did not show an association with urinary alterations in another study ( 6 ). We also included MRI findings. The major anatomical sites were selected based on the Lasmar diagram ( 14 ).

All laparoscopies and DC were performed by the same multidisciplinary team, which systematically performed the surgery. Whether identified during the preoperative evaluation or intraoperatively, all suspected lesions were carefully explored. Endometriosis was histologically confirmed when endometrial glands and stroma were present on microscopic examination. The same experienced pathologists were responsible for the histological reports.

### Statistical analysis

Statistical and graphical analyses were performed using the IBM® SPSS® Statistics Standard Grad Pack 20 (NY, USA). A 95% confidence interval (CI) was used.

## RESULTS

During this period, 170 patients underwent preoperative DC, aged 22 to 54 years (median age of 36 years). Thirteen patients were excluded because they had pelvic prolapse or previous pelvic surgeries. The demographic data are shown in Table-1 .

**Table 1:** Demographic data of the patients.

					Partner				Ethnicity			Schooling			Smoking	
	Age (Years)	Height (cm)	Weight (kg)	BMI	Married	Divorced	Single	Widow	White	Mixed	Black	<12th grading	Graduated	Post graduated	Non-smoking	Smoker
Min	22	153	45	18												
P25	32	160	60	22.5												
Median	39	164	65	24.1												
P75	45	167	70	26												
Max	54	178	108	40.2												
N					109	13	34	1	78	65	14	35	97	25	156	1
Percentage (%)					69.43	8.28	21.66	0.63	49.68	41.4	8.92	22.29	61.78	15.92	99.36	0.64
CI 95%					61.83-76.10	4.9-13.65	15.93-28.73	0.64-4.11	41.96-57.42	33.99-49.22	5.39-14.41	16.49-29.42	53.99-69.02	11.02-22.45	96.48-99.89	0.11-3.52

Min: Minimum; P25: Percentile 25; P75: Percentile 75; Max: Maximum; CI: Confidence interval; BMI: Body mass index.

Among the 157 patients included, 41 (26.1% of all sample) had abnormalities in the DC. Of these, 39 (95.12%) had BE confirmed intraoperatively and 38 (92.8%) were confirmed by histopathology. Of the 41 patients with DC lesions, 31 (75.6%) underwent partial cystectomy and 8 (19.5%) underwent bladder shaving.

When the DC findings were divided, 13 (31.7%) patients had type 1 lesions. Among these patients, 6 (46.1%) underwent bladder shaving and 6 (46.1%) underwent partial cystectomy. One patient (7.69%) showed normal laparoscopic findings. In contrast, 28 (68.29%) had type 2 lesions. In this group, 25 (89.29%) underwent partial cystectomy and 2 (7.14%) underwent bladder shaving. The other 1 patient (3.57%) had normal laparoscopic findings.

**Figure 3:**
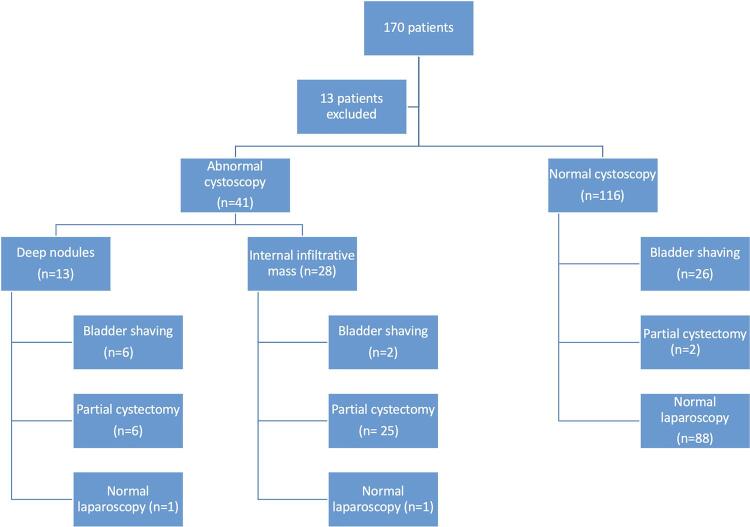
Flowchart highlighting the main findings in the sample. Thirteen patients were excluded because they had pelvic prolapses and previous pelvic surgeries. BE: Bladder endometriosis.

In the group with normal DC (116 patients, 73.89% of all sample), 28 (24.14%) had BE confirmed intraoperatively and 20 (17.24%) had histopathological confirmation. Of these 28 patients, 26 (92.86%) underwent bladder shaving and 2 (7.14%) underwent partial cystectomy. Figure 3 summarizes the DC findings.

In our sample, diagnostic odds ratio (OR) of DC was estimated by 61.28 (13.9-270.1 CI 95%) for bladder involvement and subsequent surgery by any technique. Patients with BE type 2 had a higher rate of partial cystectomy than those with BE type 1 lesions (OR 9.72 CI 95% 1.9-49.1). Using laparoscopic findings as the gold standard, DC had a calculated sensitivity and specificity of 58.21% (46.27 - 69.26% CI 95%) and 97.78% (92.26 - 99.39% CI 95%), respectively. Furthermore, predictive positive value (PPV) and negative predictive value (NPV) were 95.12% (90.54-97.54% CI 95%) and 75.86% (68.60-81.88% CI 95%), respectively.

Among the 41 patients with abnormalities in DC, 5 had a normal MRI evaluation. Among this subgroup, four patients underwent laparoscopic confirmation and bladder resection (partial cystectomy or shaving). Histopathological analysis was confirmed in all resected specimens.

In contrast, in the normal DC group, MRI detected 14 more patients with bladder changes, and 11 had laparoscopic confirmation and bladder resection. Histopathological analysis was confirmatory in 8 of the patients.

## DISCUSSION

Performing a DC rather than conventional cystoscopy is intended to increase the accuracy of BE type 1 lesions. In our study, this method had lower sensitivity but higher specificity for BE. It also has a high diagnostic odds ratio (DOR). Thus, the presence of the disease is very likely in front of a positive test result. DC may still not detect BE in many females with peritoneal or muscular diseases.

During DC, the findings can be decisive in predicting the surgical approach. Vaginal palpation associated with cystoscopy is important for assessing the disease or areas of adhesions located in the vesico-uterine septum. It is also possible to identify deep nodules in the posterior bladder wall and visualize their distance from the ureteral orifices. Palpation may also reveal thickening of the round ligaments and other indirect findings of uterine/bladder retraction. Endometrial lesions far from the ureteral orifices are simpler to resect, and those closer (<2 cm) usually require an ureteroneocystostomy ( 15 ).

BE findings vary according to menstrual cycle. During the menstrual period, an elevated area can be detected with surrounding edema. Small translucent or bluish-looking cysts can be observed due to the accumulation of blood. In the intermenstrual period, the tumor regresses in size, and only a few cysts can be seen ( 16 ).

Diagnosis through deep bladder biopsy is poorly performed because of the potential risk of bleeding and perforation. Additionally, the sensitivity is approximately 26% ( 17 ) as the lesion develops from the serosa towards the mucosa ( 4 ). In our practice, we did not perform this procedure.

However, the efficacy of cystoscopy remains unclear. A recent systematic review of BE recommended the non-routine use of this method but with a level of evidence of IV ( 4 ). Tardieu et al. concluded that no study has prospectively evaluated the diagnostic performance of cystoscopy for BE ( 9 ).

Thonnon et al. compared the effectiveness of TVUS, MRI, and cystoscopy for identifying and characterizing BE. Despite the small number of patients (n = 8), TVUS and cystoscopy were able to identify all the cases. Both methods were equivalent in measuring the distance between the lesions and the ureteral orifices ( 18 ).

A more recent study argued that TVUS alone would be able to diagnose BE and calculated an accuracy of 95% for the method compared with conventional cystoscopy. Of the 22 patients studied, TVUS identified 9 patients with BE without involvement or protrusion in the mucosa, and therefore, had normal findings on cystoscopy ( 19 ).

DC abnormalities were associated with a high OR for the need for any bladder surgery, estimated to be 61.28. The extent of transmural involvement seems to be a significant predictor of the need for partial cystectomy, since almost all women (89.29%) with BE type 2 required this more aggressive approach, with an estimated OR of 9.72, compared to BE type 1.

These data may be important, as they may predict greater surgical complexity and the need for an experienced urologist to perform a partial cystectomy or even ureteral reimplantation.

Compared with MRI, this method did not detect five patients with abnormalities in DC but identified 14 patients with BE and normal DC. Considering that MRI, in addition to TVUS, seems to have greater sensitivity and is a non-invasive diagnostic method, it seems reasonable to infer that these methods should be used preferentially instead of DC in the initial preoperative evaluation. These methods have the advantage of simultaneously identifying lesions in other structures. MRI is also intended to estimate the depth of lesions in the bladder wall and, therefore, may also be a good predictor of the need for partial cystectomy. However, there is still no standardization for this type of assessment. It should be noted that the estimation of the distance to the ureteral ostia by imaging examinations can be underestimated, depending on the degree of bladder depletion. In addition, a standard urine volume at which all patients will undergo MRI has not been established ( 20 ). Performing an MRI with a completely empty bladder allows better visualization of the vesico-uterine recess, which is one of the most affected sites ( 7 ). In contrast, MRI with a full bladder allows the assessment of bladder lesions and ureteral involvement ( 21 ).

It is important to remember that in this historical series, with a better understanding of the disease and improvement in diagnostic methods, especially the use of MRI, cystoscopy has had more restricted use over the years, as described in our methods. Thus, we may face a bias.

However, by definition, the sensitivity and specificity of a test do not vary according to pre-test probability. Thus, they can be used in different populations and to compare the diagnostic potential of different tests. On the other hand, other variables such as PPV and NPV are dependent on the prevalence of the disease; therefore, they cannot be generalized for patients with a profile different from that of the study and do not allow comparison between different diagnostic tests ( 22 ). Another limitation of our study is that it is not a prospective study. However, we used a reliable database system in which preplanned data collection was systematically performed for several years.

Therefore, in patients with lesions suspected of endometriosis on initial imaging, we recommend that DC should be performed as a preoperative strategy, considering that their findings can help the multidisciplinary team, especially the urologist, in choosing the best approach for BE.

## CONCLUSIONS

Dynamic cystoscopy seems to be a highly specific test (97.78%) at the expense of lower sensitivity (58.21%). This method also had a high positive predictive value (95.12%) and a negative predictive value of 75.86%. However, cystoscopy abnormalities are associated with a higher ratio (OR 61.28) of bladder surgery for the treatment of deep endometriosis, and BE type 2 seems to be associated with a greater ratio (9.72) of partial cystectomy, and these findings may change surgical strategy.
